# Risk-communication capability for public health emergencies varies by community diversity

**DOI:** 10.1186/1756-0500-1-6

**Published:** 2008-03-07

**Authors:** Elena Savoia, Michael A Stoto, Paul D Biddinger, Paul Campbell, Kasisomayajula Viswanath, Howard Koh

**Affiliations:** 1 Center for Public Health Preparedness, Division of Public Health Practice, Harvard School of Public Health, 677 Huntington Avenue, Boston 02115 MA, USA; 2 Georgetown University School of Nursing and Health Studies, 3700 Reservoir Road, NW Washington, DC 20057-1107, USA; 3 Massachusetts General Hospital, Department of Emergency Medicine, 0 Emerson Place, Suite 340, Boston, MA 02114, USA; 4 Dana Farber Cancer Institute, 44 Binney Street, Boston 02115 MA, USA

## Abstract

**Background:**

Public health emergencies heighten several challenges in risk-communication: providing trustworthy sources of information, reaching marginalized populations, and minimizing fear and public confusion. In emergencies, however, information may not diffuse equally among all social groups, and gaps in knowledge may increase. Such knowledge gaps vary by social structure and the size, socioeconomic status, and diversity of the population. This study explores the relationship between risk-communication capabilities, as perceived by public officials participating in emergency tabletop exercises, and community size and diversity.

**Findings:**

For each of the three communication functions tested, risk-communication capabilities are perceived to be greater in communities with fewer then 10% of the population speaking a language other than English at home, decreasing as the percentage grows to 20% (ANOVA P ≤ 0.02). With respect to community size, however, we found an N-shaped relationship between perceived risk communication capabilities and population size. Capabilities are perceived highest in the largest communities and lowest in the smallest, but lower in communities with 20,000–49,999 inhabitants compared to those with 2,500–19,999.

**Conclusion:**

The results of this study suggest the need to factor population diversity into risk communication plans and the need for improved state or regional risk-communication capabilities, especially for communities with limited local capacity.

## Findings

Public health emergencies heighten several challenges in risk-communication: providing trustworthy sources of information, reaching marginalized populations, and minimizing fear and public confusion [1]. In emergencies, however, information may not diffuse equally among all social groups, and gaps in knowledge may increase. Such knowledge gaps vary by social structure and the size, socioeconomic status, and diversity of the population [2-4]. Little is known about the factors that influence public health systems' ability to ensure that all groups are reached. This study explores the relationship between risk-communication capabilities, as perceived by public officials participating in emergency tabletop exercises, and community size and diversity. The primary research question is whether the community characteristics that influence knowledge gaps are also related to the communication capacities of public health systems.

The study population consisted of 133 individuals participating in three tabletop exercises conducted by the Harvard School of Public Health Center for Public Health Preparedness in Massachusetts and Maine in 2005 and 2006. All exercises were designed and led by faculty and staff of the Harvard School of Public Health Center for Public Health Preparedness (HSPH-CPHP), and planned in conjunction with the state of Massachusetts Health Department coalition of regional local town and city health departments and boards. The central goal of the exercise was to provide an opportunity for each region or county to test its community-wide emergency response plans, and to determine how local government agencies and community groups will work together to respond to a mass casualty event such as pandemic influenza. Participants included local, regional, and state-level professionals from a variety of disciplines such as public health, health care, law enforcement, fire services, emergency medical services, emergency management, and government. In advance of each exercise, participants were divided into small groups of 8 – 10 individuals who convened around a table. The members of each small group were chosen within regions and communities such that members of the same or neighboring communities were seated together. The exercise scenario opened with the announcement of the first human case of Avian Influenza Type A (H5N1) in the U.S., resulting in widespread infection throughout the state. The scenario spanned a timeline of 14 days in approximately 4 hours, and was designed to test a range of local and regional capabilities, including: unified command, regional coordination, inter- and intra-agency communications, risk-communication to the public, infection control, surge capacity, and mass care. Participants engaged in several risk-communication activities (using interpersonal communication, e-mails and cell phones) including designating roles and responsibilities within each agency, activating communication channels, and developing communication strategies and messages.

To assess risk-communication capabilities, participants were asked, at the end of the exercise, to rate their public health system's ability 1) to communicate with the public about up to date outbreak information, disease control requirements, individual risk reduction, when and where to seek for care, 2) to minimize fear, and 3) to reach marginalized populations through trusted sources. Responses were on a Likert scale, ranging from 1 (insufficient) to 3 (exceeds expectations). We chose two characteristics of community diversity: population size and percentage of non-English speakers to study the relationship between public health officials' perceived risk-communication ability and community diversity.

The study sample included 133 representatives of 55 communities, grouped by population size: (<2500 (n = 11), 2500–19,999 (n = 58), 20,000–49,999 (n = 40), and ≥ 50,000 (n = 24)) and by percentage of non-English speakers: (low: <10% (n = 55), medium: 10–20% (n = 30) and high: >20% (n = 48)) [5-7]. Mean values and 95% confidence intervals (C.I.) for each item were displayed graphically and differences tested by ANOVA. Community population size ranged from 603 to 94,304 residents with a median of 18,560.

**Figure 1:**
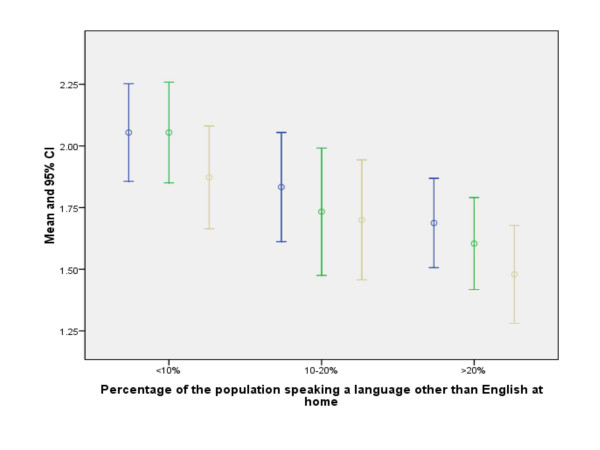
**Risk-communication capability and percentage of non-English speakers**: participants were asked at the end of the exercise to rate their community's ability to: (1) communicate with the general public about up to date outbreak information, disease control requirements, individual risk reduction, when and where to seek medical care [*blue line in graph*]; (2) communicate with the public to minimize fear [*green line in graph*]; (3) communicate with marginalized population groups through trusted sources [*beige line in graph*]. Responses were on a Likert scale, ranging from 1(insufficient) to 3(exceeds expectations). Mean and 95% C.I. are displayed for individuals grouped by the percentage of the population in their communities speaking a language other than English at home.

**Figure 2:**
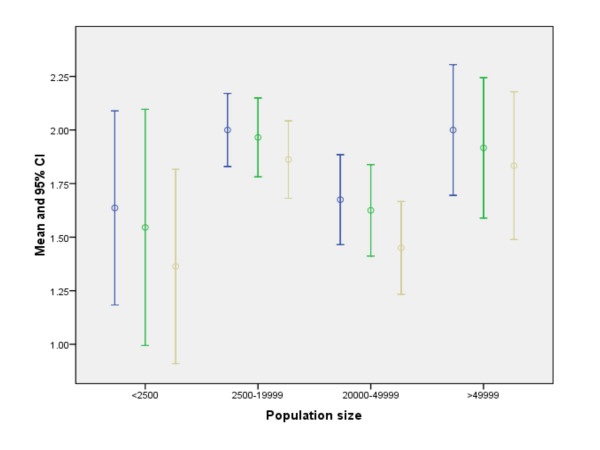
**Risk-communication capability and population size**: participants were asked at the end of the exercise to rate their community's ability to: (1) communicate with the general public about up to date outbreak information, disease control requirements, individual risk reduction, when and where to seek medical care [*blue line in graph*]; (2) communicate with the public to minimize fear [*green line in graph*]; (3) communicate with marginalized population groups through trusted sources [*beige line in graph*]. Responses were on a Likert scale, ranging from 1(insufficient) to 3(exceeds expectations). Mean and 95% C.I. are displayed for individuals grouped by the population size of their communities.

For each of the three communication functions, Figure 1 indicates that risk-communication capabilities are perceived to be greater in communities with fewer then 10% of the population speaking a language other than English at home, decreasing as the percentage grows to 20% (ANOVA P ≤ 0.02). With respect to community size, however, we found an N-shaped relationship between perceived risk communication capabilities and population size. As seen in Figure 2, perceived capabilities are highest in the largest communities and lowest in the smallest, but lower in communities with 20,000–49,999 inhabitants compared to those with 2,500–19,999. The 95% C.I. and ANOVA (P ≤ 0.05) confirm the pattern is unlikely to be attributable to chance.

## Discussion and Conclusion

An innovative aspect of this study is the assessment of factors related to emergency risk-communication inequalities in tabletop exercises. Since public health emergencies are rare, tabletop exercises that mimic emergencies can be used to provide a "next best" means of assessing public health emergency response capabilities [8,9]. While identifying the best tools to measure the quality of performance in these exercises is an evolving science, self-assessment is a useful measure of preparedness. Although the self-assessments may be biased, all exercises were assessed using the same scenario, so the information bias may be consistent across communities.

The relationship between risk-communication capabilities and population diversity is consistent with the literature cited; communication inequality arises where there are differences among social groups in the generation, management and distribution of information [10]. Inequalities in communication needs are often not usually taken into account when developing risk-communication plans. The results of this study are a call to action: communities with the greatest diversity may have the greatest need for well-trained officials and highly developed systems for risk-communication.

The complex relationship with population size may reflect the interplay of two factors. The relative lack of risk communication capabilities in small communities (<2500 population) may reflect the limited public health infrastructure in small towns, typically consisting of one part-time health official. Larger communities (≥ 50,000 population), have a more developed public health infrastructure including specialized public relations personnel [11]. On the other hand, larger communities tend to be more diverse, and this effect may overcome more limited differences in the public health infrastructure between the two middle-sized groups of communities. These results suggest the need to improve state or regional risk-communication capabilities, especially for communities with limited local capacity.

## Competing interests

The authors declare that they have no competing interests.

## Authors' contributions

ES conceived the study and performed data analysis, VV, HK, PDB, and PC provided critical revision of the manuscript for important intellectual content, MAS supervised the study. All authors read and approved the final manuscript.
